# Supplementary material for: Romantic partner embraces reduce cortisol release after acute stress induction in women but not in men
Source: PLoS One. 2022 May 18;17(5):e0266887. doi: 10.1371/journal.pone.0266887 (PMC9116618; doi:10.1371/journal.pone.0266887)
Supplement: S1 Table — Both R2 and Cohen’s f are given as effect size measures. (DOCX) [file pone.0266887.s001.docx]

|  | Cortisol | Systolic BP | Diastolic BP | Positive Affect | Negative Affect |
| --- | --- | --- | --- | --- | --- |
| Effect size without interactions | R² = 0.164  f = 0.44 | R² = 0.388  f = 0.80 | R² = 0.237  f = 0.56 | R² = 0.038  f = 0.20 | R² = 0.049  f = 0.23 |
| Effect size with interaction | R² = 0.206  f = 0.51 | R² = 0.388  f = 0.80 | R² = 0.239  f = 0.56 | R² = 0.055  f = 0.24 | R² = 0.062  f = 0.26 |
| Interaction effect alone | R² = 0.042  f = 0.21 | R² = 0  f = 0 | R² = 0.002  f = 0.04 | R² = 0.017  f = 0.13 | R² = 0.013  f = 0.11 |
