# Supplementary figures and images for: Romantic partner embraces reduce cortisol release after acute stress induction in women but not in men
Source: PLoS One. 2022 May 18;17(5):e0266887. doi: 10.1371/journal.pone.0266887 (PMC9116618; doi:10.1371/journal.pone.0266887)

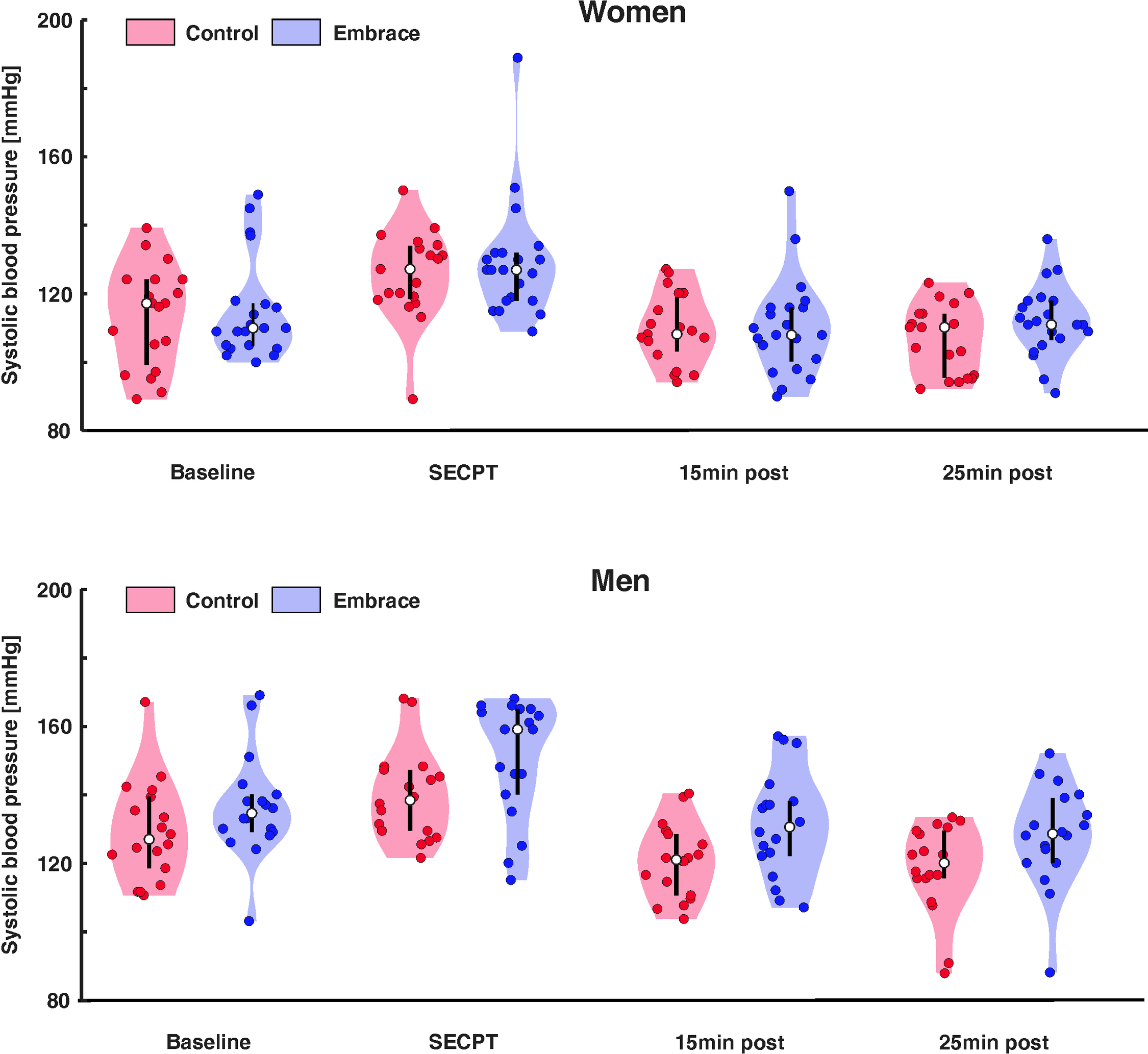

Supplement: S1 Fig — Systolic blood pressure for women (top) and men (bottom) during baseline, the SECPT and 15 minutes as well as 25 minutes post SECPT for the embrace and control condition. White dots represent the median value for each group. Error bars represent the upper and lower quartiles. (TIF) [file pone.0266887.s002.tif]

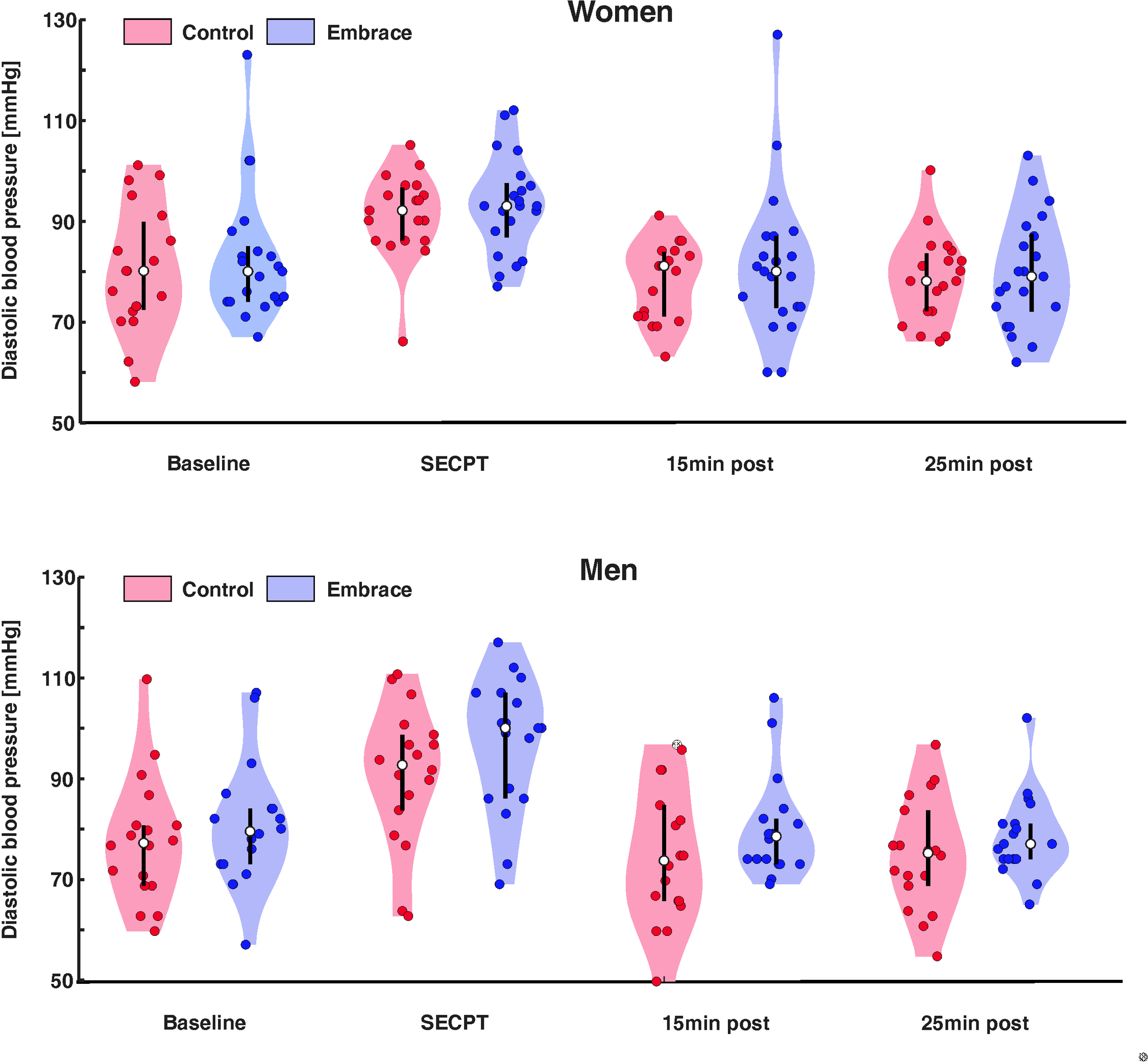

Supplement: S2 Fig — Diastolic blood pressure for women (top) and men (bottom) during baseline, the SECPT and 15 minutes as well as 25 minutes post SECPT for the embrace and control condition. White dots represent the median value for each group. Error bars represent the upper and lower quartiles. (TIF) [file pone.0266887.s003.tif]

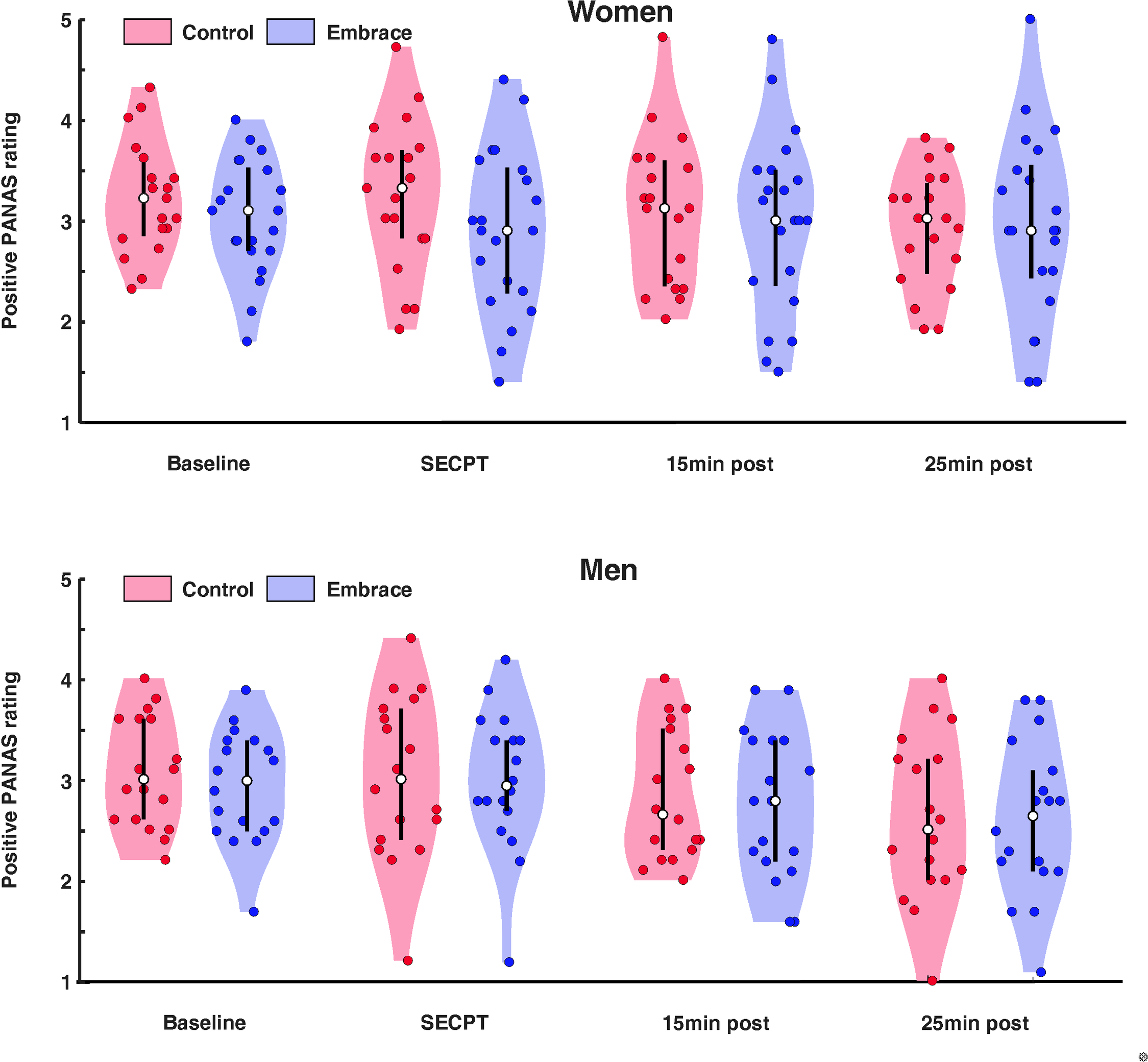

Supplement: S3 Fig — Positive affect ratings for women (top) and men (bottom) during the baseline, SECPT and 15 minutes as well as 25 minutes post SECPT for the embrace and control condition. White dots represent the median value for each group. Error bars represent the upper and lower quartiles. (TIF) [file pone.0266887.s004.tif]

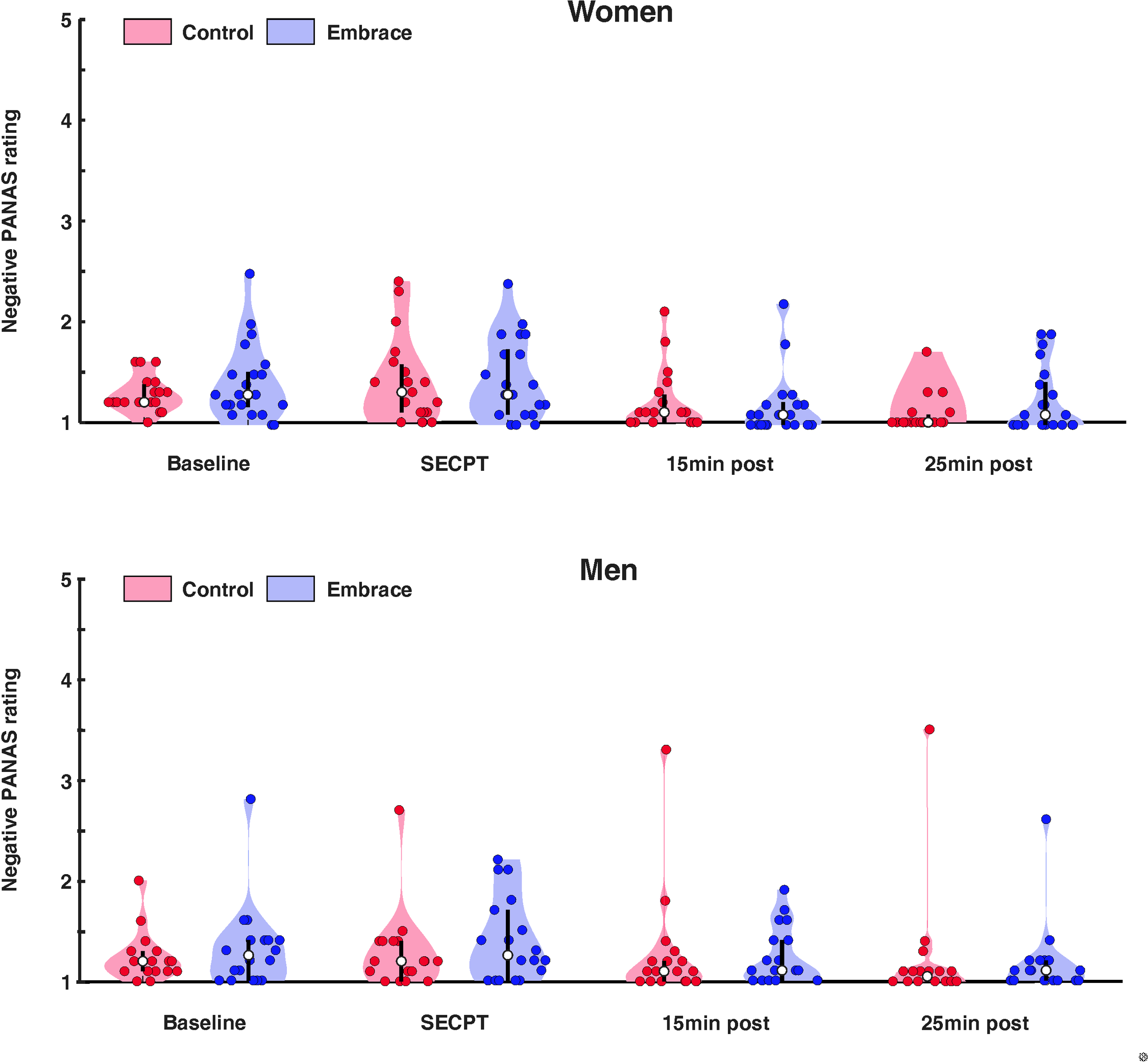

Supplement: S4 Fig — Negative affect ratings for women (top) and men (bottom) during the baseline, SECPT and 15 minutes as well as 25 minutes post SECPT for the embrace and control condition. White dots represent the median value for each group. Error bars represent the upper and lower quartiles. (TIF) [file pone.0266887.s005.tif]
